# Young athletes’ mental well-being is associated with smartphone social networking application usage and moderated by performance level and app type

**DOI:** 10.1038/s41598-024-77418-2

**Published:** 2024-11-07

**Authors:** Radha Fiedler, Jahan Geber, Markus Reichert, Michael Kellmann

**Affiliations:** 1 Faculty of Sport Science, Department of Sport Psychology, Ruhr University Bochum, Gesundheitscampus-Nord 10, 44801 Bochum, Germany; 2 Faculty of Sport Science, Department of eHealth and Sports Analytics, Ruhr University Bochum, Gesundheitscampus-Nord 10, 44801 Bochum, Germany; 3 School of Human Movement and Nutrition Sciences, The University of Queensland, Blair Drive, St Lucia, QLD 4072 Australia; 4 Department for Sport and Exercise Science, Paris Lodron University Salzburg, Salzburg, Austria

**Keywords:** Digital media, Smartphone usage, Instagram, TikTok, Youth sport, Mental health, Human behaviour, Risk factors

## Abstract

Smartphones and social media have become an integral part of human daily life and they influence mental well-being. These accounts have been researched in the general population, but not in athletes. This is critical given enhanced physical and emotional stressors in athletes versus non-athletes. Therefore, we here studied intra- and interindividual relationships of four social media sites (WhatsApp, TikTok, Instagram, and Snapchat) with mood, stress, recovery, and sleep using log-based media usage tracking as an objective measure in 53 competitive athletes aged 12–27 years. Multilevel modeling revealed that intraindividual TikTok usage negatively predicted sleep (*β* = −0.10, *p* = 0.040) and recovery (*β* = −0.15, *p* < 0.002), and positively predicted stress (*β* = 0.12, *p* = 0.018). Interindividual Instagram usage predicted feelings of calmness (*β* = 0.27, *p* = 0.029) and valence (*β* = 0.20, *p* = 0.043). Intriguingly, competition level moderated the usage outcomes of all four apps: Athletes on low performance levels showed lower mental well-being when using social media longer, yet in national and international athletes the strength of these relationships was weaker or even reversed. Our study unravels social media usage associations with mental well-being to vary as a function of performance level and provides findings on intra- and interindividual effects of critical importance to inform future population-tailored and expedient interventions.

## Introduction

Digital media impact mental health in adolescents and young adults alike^1^. In a meta-analysis, Vidal et al.^2^ reported an association between quantity of media usage and depressive mood in adolescents. Likewise, time spent on social media predicted higher social and physical anxiety, as well as depressive mood over 12 months^3^. In young athletes, digital media interact with a unique context. They face the challenge of combining several tasks and roles, e.g., school, training, recovery measures, and social life, demanding high energy and time resources^4,5^, while coping with additional stressors, e.g., pressure to perform or conflicts within a sport team. This may place athletes at a higher vulnerability of stress and mood disorders^6^. This may be particularly true for athletes on a highly competitive performance level, as in addition to positive effects of sport observed in a recreational setting, straining factors such as performance pressure and perfectionism may be increased^7,8^. Within this context, dysfunctional digital media usage may constitute an additional stressor disturbing important means of recovery, such as sleep and emotional self-regulation^9,10^. Therefore, it is vital to gain an accurate insight into the relationship between mental health and smartphone activity in this population to help optimize athletes’ daily recovery and well-being. However, up to this point little is known about the relationship of mental health and digital media usage in adolescent and young adult athletes up to this point. Fiedler et al.^10^ found that mental health in 12–19 year-old athletes was decreased when they showed prolonged usage times and addiction to digital media. Particularly on high performance levels, addiction to digital media was associated with impaired sleep. Walter et al.^11^ found a relationship between social media usage and eating disorders in young athletes.

Fiedler et al.^10^ suggest the Differential Susceptibility to Media Effects Model (DSMM)^12^ as a multidimensional framework for understanding how digital media affect psychological outcomes in athletes. Originally developed outside of the sport context, the model makes two core assumptions. First, it states that media usage is influenced by the social, dispositional, and developmental context it takes place in. Within these context variables, labelled propositions, a person’s gender or age may be considered. Within the setting of sport, the model also offers the opportunity to include sport-specific context variables, such as an individual’s role and lifestyle as an athlete, that may influence both media usage^13^ and mental health^6–8^. In particular, a highly competitive or elite performance level may be associated with additional challenges, e.g., perfectionism or performance pressure, that may interact with media usage in influencing athletes’ mental health^8^. Second, the model assumes that in interaction with the propositions, digital media usage triggers specific cognitive, emotional, and physiological responses, which mediate the effects of media on psychological outcomes.

One mediating effect may be disturbed sleep^14^. Daily media usage longer than two hours decreases sleep duration in adolescents, mediated by behavioral (bedtime procrastination due to conflicting goals) and psychophysiological reactions (disrupted circadian rhythm, increased emotional arousal) to media usage^15^. Although sleep is highly relevant for their recovery, performance, and health, prevalence of sleep disturbances in athletes are high^16,17^. In adolescents, neurological development may exacerbate sleep disturbances^18^. Taking the role of a mediator, sleep impairments related to digital media lead to increased stress, negative emotionality, and anxiety, while lack of sleep decreases the ability to cope with daily stressors and challenges^16^.

Apart from sleep, social media may impact athletes’ mood, self-esteem, and anxiety^19,20^. One effect playing an important role in this relationship is social comparison, both regarding physical appearance and athletic performance^13,21^. This may affect young athletes in particular, as comparison to peers and role models gains importance throughout adolescence^18^. Further, quality of media usage may impact mood. When experienced as fulfilling e.g., through communication or emotional support, it improves mood^22^. However, if experienced as negative, e.g., through confrontation with negative comments or content^23^, it may impair mood and increase stress.

According to the heuristic scissors model of the interrelation of stress states and recovery demands^24,25^, physical and mental stress needs to be compensated through adequate recovery activities. Limited resources (e.g., time) set a vicious cycle in motion: if the athlete is under increased stress and unable to fulfil the increased recovery demands, they experience even more stress. People can be so stressed that they do not find or take time to recover adequately or think of better ways to cope with the situation^26^. Therefore, if stress is increased through dysfunctional media usage in athletes, it can be expected that recovery effects are reduced, as they do not sufficiently compensate for the mental and physical stress the athlete experiences. Factors such as bedtime procrastination or time conflicts through an overuse of digital media may disturb recovery measures^15,17^. Again, recovery may be unsuccessful and cause negative emotions if the activity is evaluated as unfulfilling^9^.

Previous studies revealed cross-sectional relationships between digital media and mental health in the general population, young adult, and adolescent athletes^1,10,19^. However, these studies yielded heterogenous results and oftentimes small effect sizes. In part, this may be attributed to methodological issues. First, effects of social media in athletes may differ between social networks^19^. Rozgonjuk et al.^27^ name Instagram, WhatsApp Messenger, Snapchat, and TikTok as the most relevant platforms to young people. TikTok, Instagram, WhatsApp, and Snapchat all include direct messaging and the possibility to upload visual, user generated content with temporary visibility (24 h). While WhatsApp and similar apps (e.g., Signal, Telegram) include limited functions to upload permanently visible content, they do not focus on it. On Instagram, TikTok, and Snapchat the feature is more prominent. Additionally, these three platforms include a feed of suggested content personalized by an algorithm, that is not available in WhatsApp or other messengers.

Instagram holds a special position for branding and self-presentation in sports^28^. However, particularly comparison with others on this platform has been associated with negative body image and disordered eating behavior^29^. While Instagram’s start page prompts users to interact with content of users they subscribe to, the focus of TikTok is on the feed of personalized content created by the site’s algorithm instead^39^. Montag et al.^31^ suggest this leads to stronger immersion into the site. This may increase addictive potential as a driving factor of negative associations between media usage and mental health^32^. Montag et al.^31^ add that TikTok centers interaction between the user and the algorithm, instead of an interaction between users. As communication and emotional support through social networks is associated with improved mental health^22^ this may be another problematic aspect about TikTok. This suggests TikTok may have a higher risk of detrimental effects on well-being than the other platforms.

When measuring media usage, the most common measure is self-reported data. However, Ellis et al.^33^ criticize that self-reported smartphone usage has a risk of being unreliable. As an alternative, technology-based approaches like smartphone activity log-tracking can be utilized^34^. However, being relatively resource-intensive to implement, few studies have applied log-based usage tracking to examine the relationship between mental health and smartphone usage yet. This problem is particularly prevalent in research on competitive and elite athletes, where the few studies examining the relationship between digital media and mental health in athlete populations either relied on measures of self-report^10,19^ or followed a qualitative approach^13^. In addition, their cross-sectional design holds the risk of falsely interfering from group level data to an individual^35^. A prominent example depicts this well-known issue of drawing within-person inference from cross sectional data, coined “ecological fallacy”: For instance, cross-sectional studies demonstrate that individuals who engage in higher levels of physical activity tend to have consistently lower blood pressure, indicating a negative correlation between subjects. However, this design would not show a positive within subject correlation that may be expected since acute physical activity, like stair climbing, can cause an increase in blood pressure^36,37^. As Reichert et al.^38^ point out this may explain mixed findings in psychological studies—for an in-depth discussion see^36,39–41^. However, to plan effective interventions, it is necessary to properly distinguish between effects on group and individual level. In the context of smartphone usage and mental health, this means distinguishing intraindividual effects from cross-sectional relationships between participants. Conceptually, intraindividual effects describe the impact of usage differences within an individual across a defined time span, e.g., hourly, daily, weekly, yearly. When referring to a short time span, they could also be understood as short-term effects of media usage. The differentiation between intraindividual and interindividual effects may be achieved using intensive longitudinal data (timely dense, e.g., daily, repeated within-subject measurements)^42^.

## Objectives and hypotheses

Although digital media and their impact on mental well-being in both athletes and the general population is a highly relevant and current topic, studies examining this topic often face methodological issues, such as a lack of detailed or objective measurements, as well as the risk of an ecological fallacy. The current study contributes to strengthening research on this topic by measuring smartphone usage in young athletes using a log-based tracking approach. Further, this study aimed to distinguish between effects of different social media applications to allow for a more precise understanding of which aspects of media usage may be beneficial or harmful to athletes, which may have substantial implications for both research and practice within this field. In a first step, the relationship between mental well-being and application usage will be analyzed from a bidirectional perspective. Based on the assumption that competitive sport, and especially the setting of elite-level sport, is associated with a distinct set of challenges and may therefore interact with media usage effects, competition level will be entered into the analysis in a second step to test for differences between competitive elite and recreational athletes. Of note, in our study and following the well-established and recognized theoretical framework of Keyes^43^, we understand mental health as a dual continuum with the interrelated, but distinguishable dimensions of mental illness and mental well-being. While mental illness refers to psychopathology, mental well-being relates to mood states, psychological needs and optimal functioning^43^. Within this model, we here investigate the core factors mood, stress, recovery and sleep taking a predominantly well-being perspective.

Therefore, this paper aims to test the following hypotheses.

1. Usage of messenger apps, Instagram, TikTok, and Snapchat predict mood, sleep, stress, and recovery intra- and interindividually. A negative relationship between application usage and mental health is expected for TikTok and Instagram, while no directional predictions can be made for Snapchat and messenger apps.
2. Mood, sleep, stress, and recovery predict usage of messenger apps, Instagram, TikTok, and Snapchat intra- and interindividually. No directional predictions are made for this relationship.
3. Competition level influences the relationships between messenger apps, Instagram, TikTok, and Snapchat with mood, sleep, stress, and recovery.

## Methods

### Design and procedure

Statistical power analyses of multi-level models heavily depend on a variety of assumptions (e.g., random slopes, covariance structure) that cannot be estimated without the final data set. Therefore, it was tested whether the aspired sample size of *N* = 50–80 participants was adequate for detecting the predicted effects based on recent simulation studies^44^. According to simulation studies, *N* = 50–80 is sufficient to find a medium minimum detectable effect size (MDES = 0.30) in a two-level model, assuming a medium random slope component and a level 1 sample size of at least 14 at a power of 80%.

The study used log-based tracking of smartphone data. Data collection took place over 16 days, including 14 days of continuously tracking participants’ smartphone activity, resulting in detailed measures for each day during this period. The length of the tracking period was chosen as a tradeoff between extensive data collection and practicability. Data was exported after the tracking period had ended. During tracking, all participants were instructed not to look at their media activity to avoid a bias in usage behavior.

**Fig. 1:**
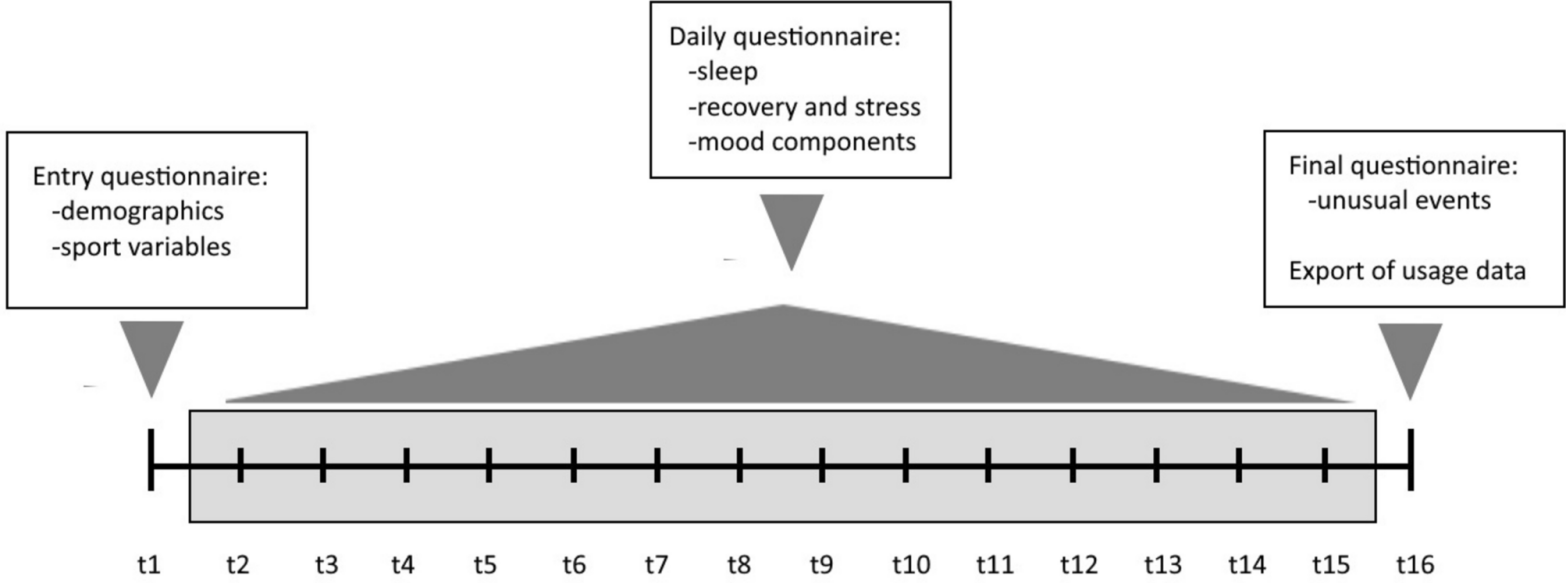
Design of the study. *Notes* t1-t16 = days of the study. Log-based tracking took place from t2-t15 (marked in grey).

One day prior to the start of the tracking period, the first questionnaire was distributed via the web app SoSci Survey (www.soscisurvey.de), asking for participants’ demographic and athletic information (e.g., gender, age, training duration, competition level). In addition, they generated a pseudonym to later match their questionnaires with their tracking data. During the tracking period, participants received a link to a daily questionnaire every morning at 5:00 a.m. with the instruction to complete it before noon. It took about two minutes to complete and contained the identical three short scales measuring the current mood, perceived quality of sleep in the previous night, as well as stress and recovery every day. On the last day, participants were asked to fill out a final questionnaire of about five minutes and were instructed to export their usage data. Data files were labeled with the pseudonym created on the first day of the study and transmitted to the researchers anonymously via the app Dropbox (www.dropbox.com). The final questionnaire included information on unusual events (e.g., prolonged sickness, competitions) during the tracking period to enable control for biased media usage behavior or mental health states. No participants needed to be excluded based on this data (Fig. 1).

### Ethical considerations

Prior to the study, all participants gave their written informed consent to participation in the study. For subjects younger than 18 years, informed consent from their parents was obtained in written form as well. The study was approved by the institutional ethical committee (Ethical Committee of the Ruhr University Bochum, Faculty of Sport Science). The research was performed in accordance with the relevant guidelines and regulations^45^.

### Sample

Initially, 58 athletes participated in the study. A convenience sample was acquired among the project’s cooperation partners (multiple sport federations, teams and clubs throughout Germany). Potential participants were informed about the study through an online flyer and in the following were able to sign up to the study. Five participants had to be excluded, as they did not fill out the initial questionnaire. As they did not generate a pseudonym their smartphone activity and self-report data could not be assigned to each other. Of the remaining 53 participants, 14 were female and 39 were male. Their age ranged from 12 to 27 years (*M* = 18.12, *SD* = 3.62). The majority of athletes played a team sport (*n* = 37), participating in either water ball, basketball, handball, or soccer. The most frequent sports in individual athletes were athletics and rowing. Following a review by Swann et al.^46^, competition level (none, informal/local, regional, supra-regional, national, and international) was chosen as a measure for athletic performance. Eleven participants reported taking part in informal/local competitions only or not competing at all. Nine participants reported competing in regional or supra-regional championships. Of the other subjects, 22 competed on a national and eleven on an international level. Athletes on national level or higher (*n* = 33) were classified as elite athletes. For the analysis performance levels were aggregated to elite (competing on national level or higher) and non-elite level (competing lower than national level). To exclude a bias through unusual events during the tracking period, participants were asked on the last day whether their training, mental well-being, or media usage differed significantly from the previous weeks. No participants needed to be excluded based on this data. The relative distribution of male and female athletes across team and individual sports, as well as across aggregated performance level was even.

### Measures

To examine daily intraindividual changes, as well as interindividual differences, measures of mental health needed to be suited as both trait and state variables. Of the variables previously indicated to relate to digital media^15,19,47^, sleep quality, mood, stress, and recovery met this criterion. Following Reichert et al.^38^, measures needed to satisfy two additional criteria. First, the instruments needed to be adequately sensitive to change and refer to the current moment, rather than to a longer period of time. Second, they needed to be very economic to avoid the daily questionnaire being too long, thereby risking the loss of participant compliance. Cronbach’s Alpha in the current study is reported over all individuals and measuring points.

### Mood

Mood was assessed by the six-item short form of the Multimodal Mood Questionnaire (MMQ)^47,48^. Originally measuring valence, energetic arousal, and calmness with twelve items, the six-item short form was validated by Wilhelm and Schoebi^49^ and comprises two items per scale. It is highly sensitive to change, making it suitable to measure current mood states. Wilhelm and Schoebi^49^ reported an acceptable Cronbach’s Alpha of α = 0.70—0.77. In the current study Cronbach’s Alpha was α = 0.814 for energetic arousal, α = 0.805 for calmness, and α = 0.831 for valence.

### Recovery and stress

The Short Recovery and Stress Scale (SRSS)^50,51^ consists of eight items and has been derived from the eight scales of the Acute Recovery and Stress Scale (ARSS)^50,51^ which were then grouped into the Short Recovery Scale [Physical Performance Capability, Mental Performance Capability, Emotional Balance, Overall Recovery] and the Short Stress Scale [Muscular Stress, Lack of Activation, Negative Emotional State, Overall Stress]. The internal consistency (Cronbach’s α) for the SRSS ranges between α = 0.78 and α = 0.84. In the manual, Kellmann and Kölling^50,51^ report good sensitivity to change, good convergent validity, and homogeneity for its two scales. In the current study Cronbach’s Alpha was α = 0.735 for stress and α = 0.857 for recovery.

### Sleep

Sleep was assessed by items of an adapted version of the Evening-Morning-Protocol^52^ and its short version^53^. The Evening-Morning-Protocol is a common measure in Germanophone sleep research that shows good validity and reliability. The adapted six-item scale has been developed by a research group in the authors’ lab as part of a screening tool for recovery and immunological stress reactions and aims to measure subjective sleep quality in the next morning particularly in adolescent and young adult athletes. While currently under evaluation, it displays good validity and reliability. In the current study, Cronbach’s Alpha was α = 0.823. The medium sized intercorrelation with recovery and stress (Table 2) indicate good criterion validity.

### Smartphone activity data

The procedure to track smartphone activity slightly differed between operating systems. Android users were provided with the app *App Usage* from the Google Play Store. This app runs in the background and allows for detailed tracking of several parameters such as time spent on an app or how often the smartphone got unlocked every day. For iOS, neither *App Usage* nor a similar app could be employed, as iOS devices do not allow for third party apps to access data on smartphone usage. Thus, smartphone activity for iOS users was measured using the data from the inbuilt *Screentime* app. This held the disadvantage that participants needed to manually screenshot their usage data to export them. Compared to *Screentime*, *App Usage* presents data in a less aggregated fashion and would have allowed for a more detailed analysis of usage parameters. However, as only few Android users participated, data analysis was limited to the parameters comparable in *Screentime* and *App Usage*.

Following Rozgonjuk et al.^27^ data was extracted for TikTok, Instagram, Snapchat, and messenger apps. The category of messenger apps included WhatsApp Messenger, as well as the structurally and functionally similar apps Signal and Telegram. While Rozgonjuk et al.^27^ also name Facebook as one of the most popular platforms, only two participants used Facebook in our sample, which is why this platform was excluded from the analysis. Further, apps were considered for analysis if used by more than 50% of participants at least once. However, no app other than the ones already extracted satisfied this condition.

### Data analysis

Data analysis was conducted in IBM SPSS v.27. Descriptive statistics were calculated for media usage and mental well-being. To analyze relationships between smartphone usage and mental well-being, multilevel linear models were applied. In the first step, psychological well-being measured in the morning of each day was predicted from the time spent on an app the previous day. In the following, these predictors will be referred to as lead values. To determine whether changes in mental health follow, or rather precede media usage, app usage of the following day was also included. In the following these predictors will be referred to as lag values. Parameters for all models were estimated using a maximum likelihood approach.

Level 1 represented intraindividual differences to examine if psychological well-being differed within an individual between days on which smartphone usage was longer or shorter. Level 1 predictors were each day’s time spent on TikTok, Instagram, Snapchat, and messengers, for both the day preceding and following the daily questionnaire. Level 1 variables were centered around the subjects’ mean to differentiate within-person from between-person effects, as well as to avoid intercorrelation between the lead and lag values. Level 2 represented interindividual differences, examining if individuals who use smartphone apps more differ in their psychological well-being from individuals who use their smartphone less. The predictors on level 2 represented the individual mean times spent using the respective apps over the whole tracking period. Mood components, sleep, stress, and recovery were included as dependent variables, resulting in a total of six models.

**Fig. 2:**
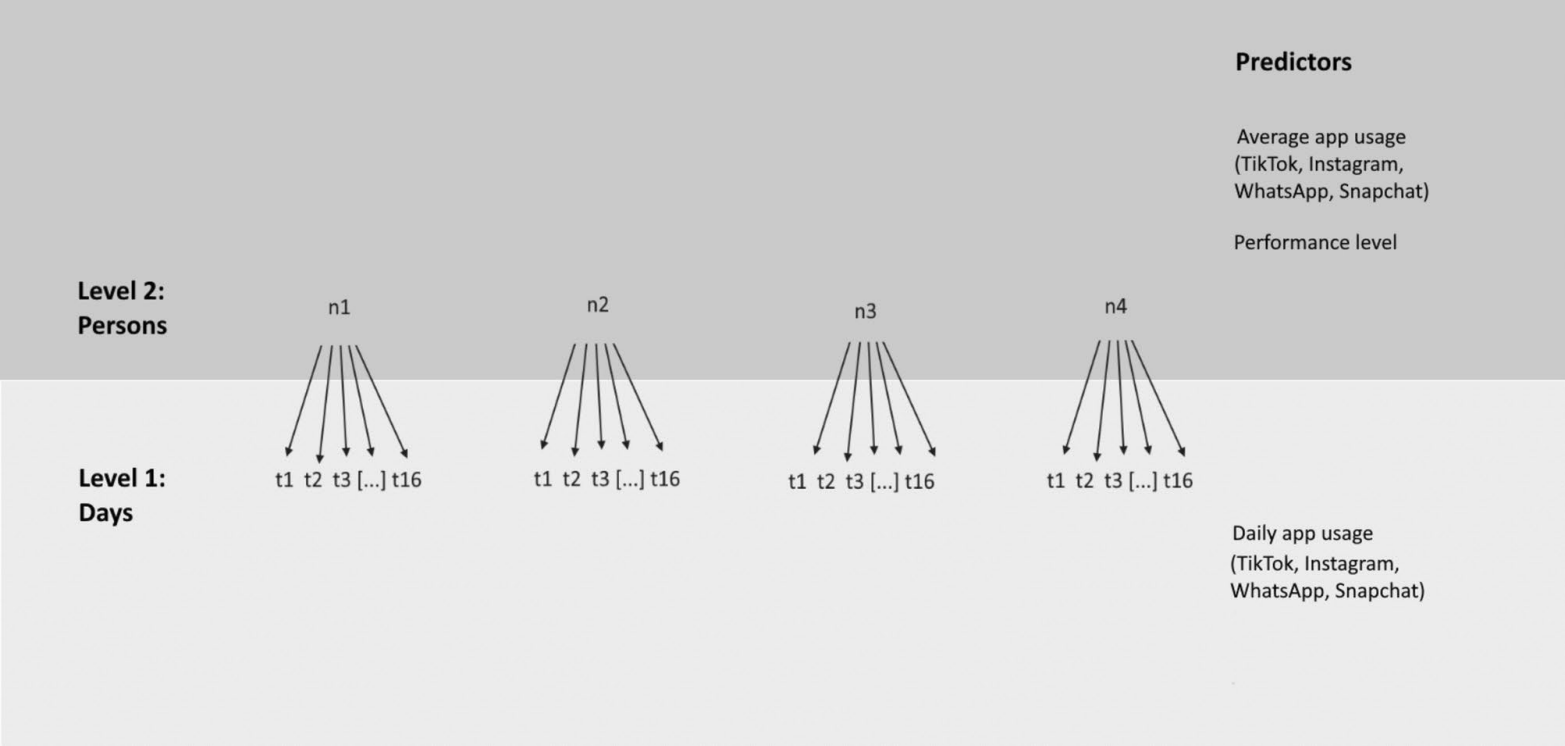
Conceptual statistical model used within the study. *Notes* t1-t16 = days of the study, n1-nx = participants in the study.

In the second step, interactions between intraindividual and interindividual app usage and participants’ performance level were tested. To keep the analysis feasible, only the interindividual means and the lead values, but not the lag values were included into this set of models. Following the suggestions of Swann et al.^46^, athletes were split into an elite performance level (those competing on a national level or higher) and a non-elite performance level (those competing lower than national level) (Fig. 2).

## Results

### Descriptive statistics

Descriptive statistics regarding digital media and mental well-being are displayed in Table 1.

**Table 1:** Descriptive statistics.

Variable (Range)	Total (*N* = 53)	Regional level (*n* = 20)	Elite level (*n* = 33)
EA (− 6.0–6.0)	1.01 (2.72)	0.79 (2.56)	1.15 (2.81)
Calmness (− 6.0–6.0)	1.15 (2.42)	1.84 (2.41)	2.35 (2.40)
Valence (− 5.0–6.0)	2.35 (2.29)	2.05 (2.40)	2.55 (2.20)
Sleep (1.7–7.0)	5.56 (1.00)	5.42 (1.06)	5.56 (0.94)
Recovery (2.0–7.0)	4.75 (1.06)	4.66 (1.06)	4.81 (1.05)
Stress (1.0–5.8)	3.10 (1.08)	3.00 (0.99)	3.16 (1.13)
Instagram (0–128.66)	40.33 (45.72)	24.12 (29.83)	56.12 (52.56)
TikTok (0–147.29)	21.40 (44.85)	26.02 (45.24)	16.90 (44.19)
Snapchat (0–389.00)	15.54 (64.15)	7.76 (24.31)	23.19 (86.53)
MA* (0–175.14)	30.30 (39.04)	27.22 (40.38)	33.30 (37.49)

Interindividual means and standard deviations (in brackets) of mental well-being and media usage variables. The category Messengers includes the apps WhatsApp, Telegram, and Signal. Usage time is presented in minutes.

*MA Messenger apps.

Out of 53 participants, 52 used a messenger app and 51 used Instagram at least once. TikTok was used by 37 and Snapchat by 44 people. One participant used neither of these four apps. Table 2 displays the intercorrelations between the study variables’ individual means. TikTok showed a negative correlation (*r* = 0.26, *p* < 0.001) with Instagram and a positive correlation to Snapchat (*r* = 0.54, *p* < 0.001). Competition level was related positively to Instagram.

**Table 2:** Intercorrelations of the study variables.

	Insta*	TikTok	Snap*	MA*	EA*	Calm*	Val*	Sleep	Rec*	Stress
Insta*										
TikTok	− 0.26**									
Snap*	− 0.07	0.54**								
MA*	0.08	− 0.01	− 0.06							
EA*	0.18**	− 0.02	− 0.06	− 0.08						
Calm*	0.27**	0.05	− 0.11	− 0.11*	0.49**					
Val*	0.29**	0.15*	− 0.17*	− 0.13*	0.59**	0.74**				
Sleep	0.00	− 0.17	− 0.02	0.07	0.48**	0.51**	0.52**			
Rec*	0.12	0.02	− 0.04	− 0.16	0.66**	0.68**	0.71**	0.71**		
Stress	− 0.17	− 0.01	− 0.10	− 0.03	− 0.53**	− 0.47**	− 0.52**	− 0.50**	− 0.70**	
PL*	− 0.45**	− 0.11*	− 0.15**	− 0.11*	0.07	0.10*	0.11*	0.11*	0.07	− 0.07

Intercorrelation between level 2 variables (individual means over the tracking period). * = *p* < 0.05. ** = *p* < 0.01.

*Insta Instagram.

Snap = Snapchat.

*MA* messenger apps (WhatsApp, Telegram, Signal).

*EA* energetic arousal.

*Calm* Calmness.

*Val*, Valence.

*Rec*, Recovery.

### Multilevel analysis

The relationship between digital media and mental well-being was examined using multilevel models. In the lead models, intraclass correlation coefficients were QI = 0.539 for sleep, QI = 0.549 for recovery, QI = 0.518 for stress, QI = 0.261 for energetic arousal, QI = 0.490 for calmness, and QI = 0.400 for valence. For example, this means that for energetic arousal 46.1% of variance (1-0.539 = 0.461) can be attributed to within-subject variance. The full parameters for each model can be found in the supplement. To ensure robustness of the results against imbalances in the sample, gender and team vs. individual sports were tested as control variables. This did not significantly alter the results.

### Analysis of main effects

#### Results for TikTok

Between participants, TikTok usage does not predict mental well-being. Within participants, TikTok negatively predicted quality of sleep (*F*(1, 209.0) = 4.29, *p* = 0.040, *B* = -0.004, CI [-0.008, -0.001], *β* = -0.10) and recovery within participants (*F*(1, 209.7) = 10.12, *p* = 0.002, *B* = -0.006, CI [-0.010, -0.002], *β* = -0.15), while positively predicting stress (*F*(1, 213.4) = 5.66, *p* = 0.018, *B* = 0.005, CI [0.001, 0.010], *β* = 0.12). Only mood components were not related to TikTok. In tendency, the lag value for TikTok was positively related to stress. However, this effect was not significant.

### Results for Instagram

Interindividual effects were found for the relationship between Instagram and calmness (*F*(1, 31.0) = 5.27, *p* = 0.029, *B* = 0.018, CI [0.002, 0.034], *β* = 0.27), as well as valence (*F*(1, 31.0) = 4.45, *p* = 0.043, *B* = 0.013, CI [0.001, 0.027], *β* = 0.20). This indicates that participants who used Instagram more had higher calmness and valence on average. A positive but insignificant tendency was observed for recovery and energetic arousal. The lag value was positively related to sleep, indicating that Instagram usage was higher following nights with good sleep (*F*(1, 201.2) = 3.89, *p* = 0.050, *B* = 0.003, CI [0.001, 0.005], *β* = 0.09). Positive tendencies occurred between the lag value and energetic arousal and valence.

### Results for Messengers

No interindividual relationships were observed between messengers and well-being. However, a positive tendency was observed for valence. The lag value was negatively related to sleep, indicating that messengers were used less following nights of good sleep (*F*(1, 195.7) = 7.91, *p* = 0.005, *B* = -0.005, CI [-0.009, -0.002], *β* = 0.12). The lead value displayed a negative, but non-significant tendency towards a relationship with stress.

### Results for Snapchat

No significant effects were found for mental well-being and Snapchat. In tendency, interindividual usage of Snapchat was negatively related to energetic arousal.

### Analysis of interaction effects

#### Results for TikTok

**Fig. 3:**
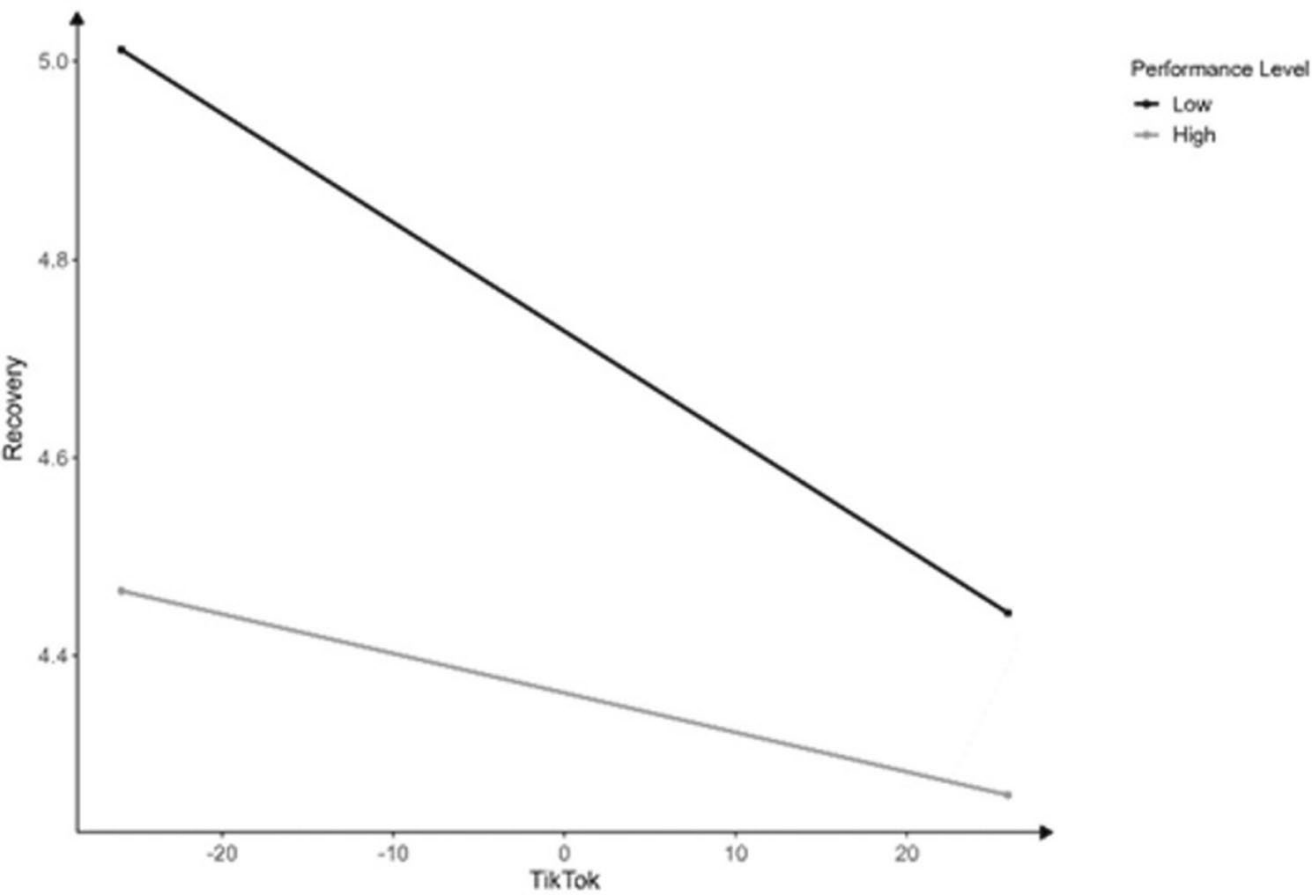
Interactions of competition level with TikTok. *Notes*: Results refer to within-subject effects.

A significant interaction was found between intraindividual TikTok usage and performance level (*F*(1, 233.1) = 5.02, *p* = 0.026, *B* = 0.007, CI [0.001, 0.014], *β* = 0.11) for recovery, indicating a slightly stronger negative relationship between TikTok and recovery on a low, than on a high performance level (Fig. 3).

### Results for Instagram

**Fig. 4:**
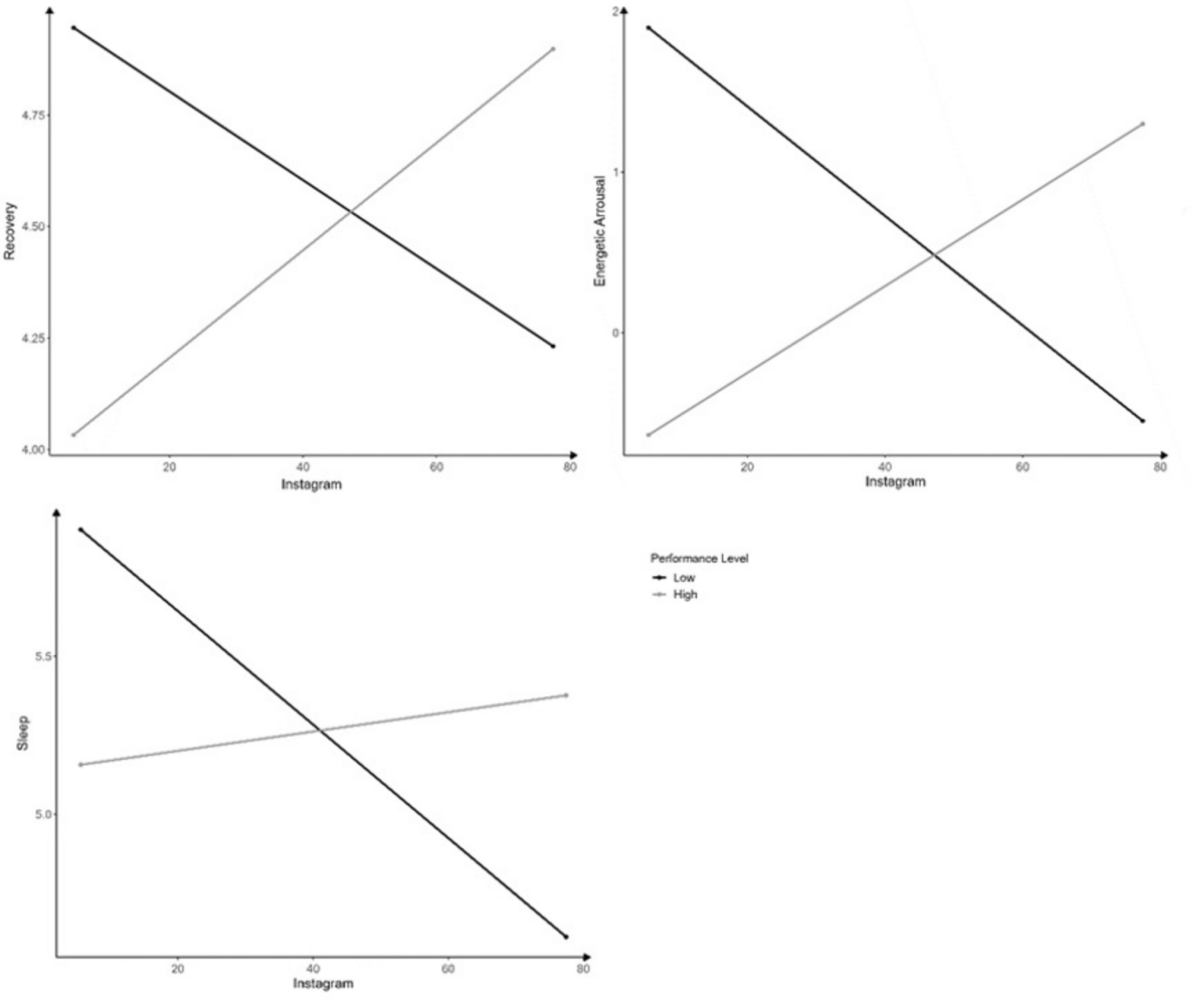
Interactions of competition level with Instagram, *Notes* Results refer to between-subject effects.

On an interindividual level, Instagram interacted significantly with performance level for sleep (*F*(1, 30.80) = 4.56, *p* = 0.041, *B* = 0.021, CI [0.001, 0.042], *β* = 0.791), recovery (*F*(1, 30.74) = 5.10, *p* = 0.041, *B* = 0.021, CI [0.001, 0.042], *β* = 0.783), and energetic arousal (*F*(1, 26.39) = 8.12, *p* = 0.008, *B* = 0.021, CI [0.017, 0.105], *β* = 0.843). This indicates a positive relationship between mental health and Instagram usage in athletes on a high performance level, while on a low performance level a negative relationship occurred. Within participants, no significant effects were found. Only a positive but non-significant tendency was observed for recovery (Fig. 4).

### Results for Messengers

**Fig. 5:**
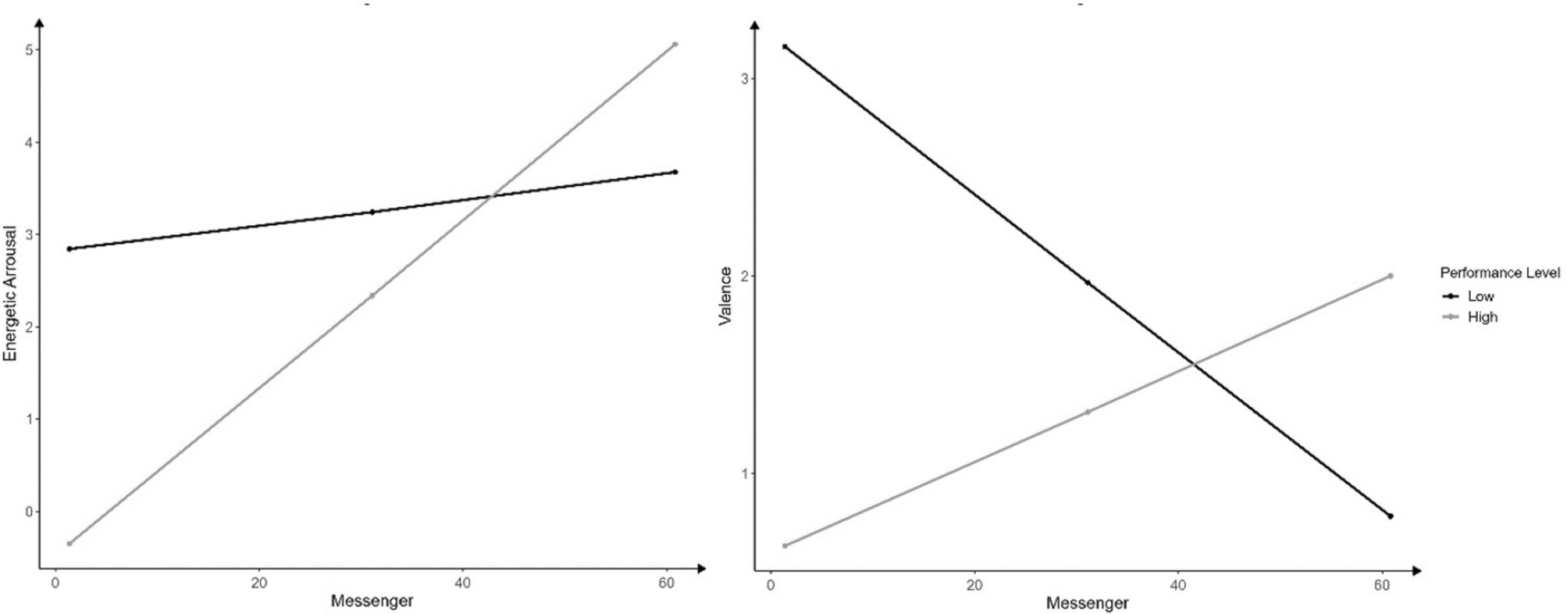
Interactions of messenger apps with mental well-being, *Notes* Results refer to between-subject effects.

Interindividual messenger usage interacted significantly with performance level for energetic arousal (*F*(1, 28.70) = 5.30, *p* = 0.029, *B* = 0.077, CI [0.009, 0.146], *β* = 0.685) and valence (*F*(1, 31.44) = 5.43, *p* = 0.026, *B* = 0.063, CI [0.008, 0.119], *β* = 0.647). In tendency, all athletes’ energetic arousal increased with messenger usage, but this effect was stronger in high- compared to lower-performance athletes. Between subjects, valence and messenger usage were related negatively in athletes on a low performance level, but positively in athletes on a high performance level (Fig. 5).

### Results for Snapchat

**Fig. 6:**
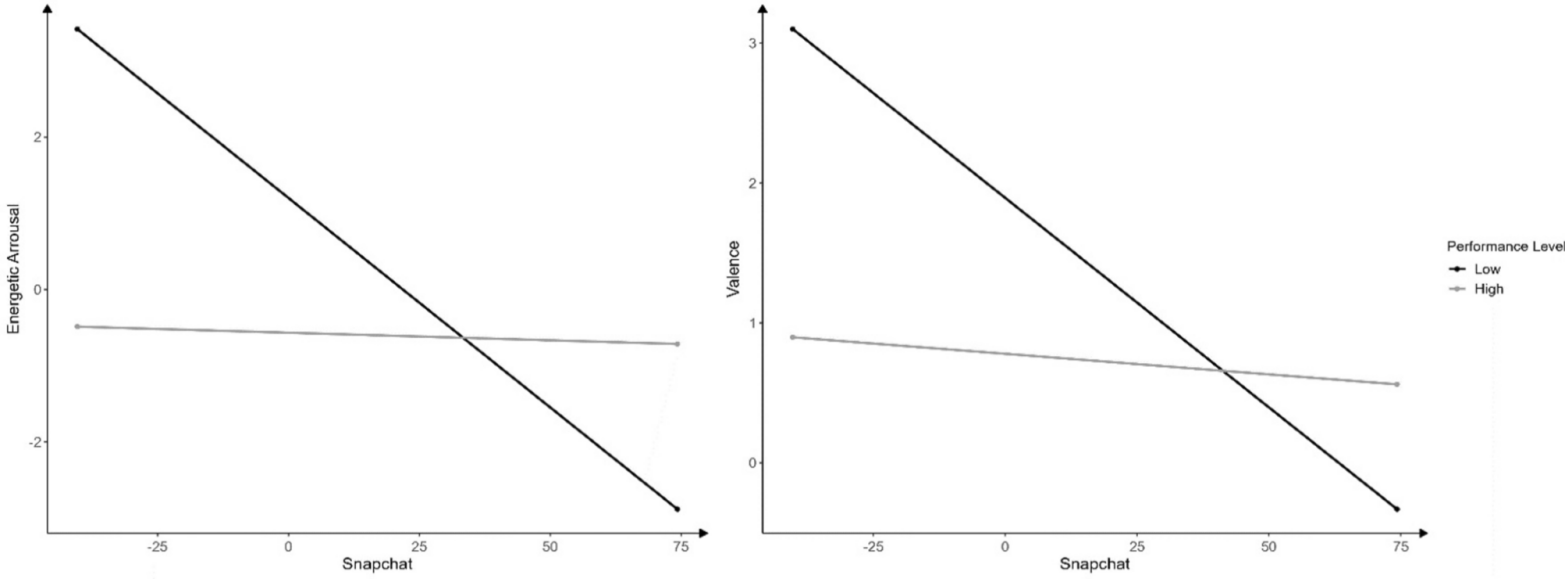
Interactions of Snapchat with mental well-being. *Notes* Results refer to between-subject effects and are not centered.

A significant interaction occurred between mean Snapchat usage and energetic arousal (*F*(1, 55.4) = 11.00, *p* = 0.002, *B* = 0.053, CI [0.001, 0.053], *β* = 0.979) as well as valence (*F*(1, 41.20) = 4.27, *p* = 0.045, *B* = 0.027, CI [0.002, 0.085], *β* = 0.592). This indicates that both athletes on a low and high performance level descriptively showed a decrement in energetic arousal and valence when using Snapchat more. However, this relationship was stronger in athletes on a low performance level (Fig. 6).

## Discussion

All three hypotheses were partly confirmed. In accordance with previous findings in athletes^10,13^, longer TikTok usage was related to impaired recovery and sleep, as well as higher stress within participants. Montag et al.^31^ suggest that unlike other social media, TikTok focuses on users’ own content, as well as a feed of personalized content selected by the app’s algorithm. When overusing TikTok, aspects of social media promoting mental health, e.g., the connection with others, may be neglected^22^. Further, high personalization and immersion into a social network might engender addictive tendencies, leading to overuse^31^ and possibly bedtime procrastination^15^. Sleep being an important form of recovery^17^, its negative association with TikTok within subjects contributes to explaining the negative relationship between TikTok and recovery.

Recovery may be viewed as a key component, as it is significantly related to TikTok across performance levels. Kellmann et al.^25^ define recovery as not solely a passive, but pro-active process determined by individuals’ decisions and directly shaping their free time. To recover successfully, it is necessary to distance oneself from previous activities and select a suitable recovery strategy. TikTok usage may disturb the step of distancing when continued in favor of a suitable strategy and in consequence hinder the recovery process^31,56^. Alternatively, TikTok itself may be selected as a recovery strategy, but may not succeed. Reasons may be inherent aspects of viewed content (e.g., confrontation with content causing negative emotions)^23^, consequences of excessive usage (e.g., exacerbated time conflicts in young athletes)^5^, or meta-cognitive evaluations of usage (e.g., anger at oneself, when past usage is experienced as a waste of time)^9^. Although these effects did not reach significance for the lag values, it is indicated there may be a positive, bidirectional relationship between TikTok and stress. This may be attributed to both indirect effects on recovery^5,9,31^, as well as on direct stress caused by TikTok through increased cognitive and emotional negative arousal^55,56^. This holds the danger of a vicious cycle, causing further increment in stress and decrement in recovery. These findings may be highly relevant to athletes aiming to optimize their recovery.

Instagram usage was positively related to calmness and valence on an interindividual level. This contradicts previous findings reporting an increment of negative affect with usage of social media in athletes^10^. An explanation may be the moderate duration of media usage observed in the current study. This would imply an inversely U-shaped, instead of a strictly linear relationship between media usage duration and mental well-being, indicating both very low and very high media usage durations may be associated with impaired mental health. From a positive perspective, Instagram may be a mean of recovery, a pass-time, or a tool to communicate with family and friends^4,22^. Seen negatively, it may be associated with addictive behavior, unfavorable social comparison, or a lack of alternative interests^10^.

Both the lag values for Instagram and messengers were significantly related to sleep of the previous day. This effect was negative for messengers, and positive for Instagram, indicating that following nights of good sleep messengers were used less, and Instagram was used more. An association with weekends or other free days may be assumed, leaving more time for leisure social media usage, but possibly also to meet others in person than through the phone and a reduced need to use messengers for functional communication (e.g., organizational purposes)^4,22^. Although its focus on one-on-one communication may have suggested the opposite^22^ no main effect was found between the lead value of messenger apps and mental well-being. Again, communication on messenger apps may not only serve the motive of connection but also be of functional or organizational nature^4^, suggesting effects of messenger usage may strongly depend on its purpose. This may also be true for Snapchat, either focusing on exchanging short videos with friends (increasing similarity to messengers) or in consuming other users’ content (increasing similarity to TikTok). For Snapchat, no significant main effects were found at all. Notably, messenger apps and Snapchat displayed shorter usage times than Instagram and TikTok, indicating they may be less prominent in young athletes’ usage behavior. For messengers, this is backed up by Rozgonjuk et al.^27^ who found WhatsApp addiction scores were lower than addiction scores to other social networking sites, which may reduce negative effects between digital media and psychological well-being^32^.

Although athletes were expected to be more vulnerable to detrimental effects of media usage on mental well-being^5,6^, the effects of all four apps’ usage were more favorable for elite, than non-elite athletes over all four social networking sites. For Instagram and messenger apps, the directions of the relationships changed, while for TikTok and Snapchat, the strength of the relationships changed depending on performance level. High-level athletes reported training longer and therefore may benefit from a protective effect of training. Mechanisms for an effect like this could either be physiological or psychological, e.g., by increased confidence through exercise^57^. A positive effect of exercise on mood may also act protectively against negative effects of media usage on mental well-being. This may be most applicable for within-subject TikTok usage and between-subject Snapchat usage, both displaying a negative relationship between app usage and mental well-being, that is significantly less pronounced in elite than in non-elite athletes, however.

Social comparison with peers and celebrities may explain both Instagram’s main and interaction effects on mood^18^. While social comparison can improve mental well-being when following the goal of self-improvement^58^, it also has been associated with impaired mental health, e.g., reduced self-esteem and negative affect in non-athletes^22^. Possibly, high-performance athletes use Instagram more functionally by seeking social comparisons motivating them to improve themselves. Additionally, the effect of social comparison on mental health is moderated by self-esteem^22^. Athletes may perceive themselves more positively compared to others on Instagram. However, this would conflict with the findings by Mancine et al.^59^ who found a higher prevalence of disordered eating in athletes than in non-athletes. Third, athletes may pursue other activities on Instagram, than their non-elite peers. Passive, rather than active usage of social networking sites is associated with impaired mental health^60^. For example, elite athletes may use Instagram more to present themselves and less to passively consume other users’ content.

Fiedler et al.^10^ found the lowest sleep quality in young elite athletes with high social media usage compared to elite athletes with shorter media usage and lower performing athletes with high media usage. The current study contradicts these findings. As mentioned previously, this may be explained with a low prevalence of excessive media usage in the current sample compared to Fiedler et al.^10^. Long media usage durations, particularly close to bedtime, may be more likely to interfere with athletes’ sleep given their narrower daily schedule^5,15^, while shorter media usage may not conflict with other demands. A similar explanation can be applied to the relationship between Instagram and recovery. Since recovery requires distancing from a previous activity to implement a selected strategy^9^, over usage of social media may interfere with recovery, while appropriate usage may not. Again, this would point towards an inversed U-shaped relationship between the length of media usage in high performance athletes regarding both recovery in general and sleep as a specific form of recovery. Assuming self-esteem, favorable social comparisons, and active, structured usage as mechanisms for a positive impact of Instagram on well-being in elite athletes, a risk would be in inversion of these effects when an athlete suffers from an injury, a major setback, or a decrement in performance. Further, particularly up-and-coming athletes who did not reach an elite level yet may be seen as a more vulnerable population to adverse effects of social media usage.

As any study, the current study holds certain limitations. Complications occurred regarding smartphone operation systems, as usage data cannot be obtained from external apps in iOS. Since iOS devices are more prevalent than Android devices in the target population, a sample including only Android users would not have been representative. In making Android and iOS data comparable, the depth of usage data that was extracted needed to be limited, excluding more detailed aspects of each app’s usage, e.g., late night activity. Further, the activities that apps are used for would be of interest, e.g., if they were used actively or passively^60^ or which motives the sites were used for (e.g., communication, self-presentation, coping). However, this information would either require more invasive tracking methods or the usage of self-report. As either of these methods may reduce the objectivity of the electronically measured data and reduce compliance, it was refrained from obtaining this information in the current study. However, future studies need to work with this tradeoff, since not only quantity, but also quality of media usage may impact mental well-being^32^.

Alongside a limited period of data availability in iOS, the more complicated procedure in iOS than in Android restricted the length of the tracking period. This shifted the choice of constructs regarding mental health to aspects that can be expected to change within short periods of time and the conceptualization of mental health towards well-being rather than a clinical perspective. Further, this limits the representativeness of the tracking period for athletes’ season. Falling into early spring, the tracking period included late competition phase for team sport athletes, and early competition phase or late preparation phase for individual sport athletes. It cannot be fully excluded that media effects may differ between competition phases. Therefore, future studies should consider tracking usage over longer periods of time to expand the understanding of the relationship between app usage and mental well-being to the clinical aspect of mental health, e.g., disordered eating. However, this requires a more efficient method of exporting data from iOS. Alternatively, multiple tracking periods taking place in each competition phase should be implemented.

The current sample displayed certain imbalances. Although the analyses showed to be robust when including these variables as covariates, it should be considered that the study may represent some athlete subgroups better than others. First, the study included more male than female athletes. This may pose a limitation as media effects may differ between genders in both athletes and the general population^62^. Specifically, Nimiya et al. (2023) conclude that Instagram usage is associated with higher body image concerns in female users, while male athletes display more exercise motivation. Although this study refers to a sample of non-athletes, this indicates that particularly the positive association between Instagram usage and mood variables in elite athletes need to be validated in female athletes. Second, team sports were represented in the sample more strongly than were individual sports. Although media effects between team sport and individual athletes have not been examined yet, they have been shown to differ in mental health [64]. Again, future studies should aim to validate the current findings in individual athletes or even draw explicit comparisons between team and individual sports.

Finally, certain relationships between media usage and well-being may also be non-linear. Moderating effects of performance level support this notion. Therefore, future studies should consider testing for non-linear effects of smartphone usage on mental well-being.

## Conclusion and practical implications

In this study, Instagram was related to higher calmness between participants, while TikTok displayed a bidirectional negative relationship with sleep, recovery, and stress within participants. The analysis of interaction effects revealed a distinct pattern between athletes competing nationally or internationally level and those on a lower performance level. Exempt a descriptively positive relationship between messengers and energetic arousal, athletes on low competition levels displayed decreased mental well-being when using either application longer. High-performance athlete’s app usage and mental health generally showed positive relationships. Only intraindividual TikTok usage was related negatively to recovery in high-performance athletes, although this relationship was weaker than in those competing on a low level.

As digital media are ubiquitous in young athletes’ lives it is essential to understand their relationship with mental well-being to improve recovery management and mental health. The current findings offer valuable insights in implying that differential approaches to interventions may be necessary depending on competition level. For example, group interventions for high-performance athletes may need to focus more on digital media’s impacts on recovery management than on mood, while in athletes on a low performance level both aspects need to be centered. When aiming to avoid negative aspects of smartphone usage on recovery, special attention should be given to the usage of TikTok. However, it should be considered that the generalizability of these results may be limited by the relatively small sample size, as well as the influence of potential moderators, such as gender or sport. Consequently, mechanisms of the relationship between media usage and mental well-being on both high and low competition levels need to be continued in future studies to identify high-risk patterns not only on a group, but also on an individual level.

## Supplementary Information

Supplementary Information.

## Data Availability

Data will be made available on request. To request the data, please contact Radha Fiedler (radha.fiedler@rub.de) or Michael Kellmann (michael.kellmann@rub.de).
