# Supplementary material for: Young athletes’ mental well-being is associated with smartphone social networking application usage and moderated by performance level and app type
Source: Sci Rep. 2024 Nov 7;14:27048. doi: 10.1038/s41598-024-77418-2 (PMC11543683; doi:10.1038/s41598-024-77418-2)
Supplement: Supplementary file 1 — Supplementary Information. [file 41598_2024_77418_MOESM1_ESM.docx]

**Supplementary Table S(1)**

*Parameters of the mixed model analyses for sleep, recovery, and stress.*

|  | **Outcome** | **Fixed B** | ***SE*** | ***F* (df)*; t* (df)** | ***p*** | **Random Estimate** | ***SD*** | **Wald-*Z*** | ***p*** |
| --- | --- | --- | --- | --- | --- | --- | --- | --- | --- |
|  | **Sleep** |  |  |  |  |  |  |  |  |
|  | Intercept | 5.688 | 0.283 | *F*(1, 31.14) = 404.18*; t*(31.14) = 20.1 | < 0.001 | 0.401 | 0.115 | 3.477 | <0.001 |
|  | Instagram (b) | -0.002 | 0.003 | *F*(1, 31.08) = 0.41*; t*(31.08) = -0.64 | 0.525 |  |  |  |  |
|  | TikTok (b) | -0.005 | 0.004 | *F*(1, 32.89) = 1.62*; t*(32.89) = -1.27 | 0.212 |  |  |  |  |
|  | Messenger (b) | 0.005 | 0.005 | *F*(1, 31.61) = 1.1*; t*(31.61) = 1.05 | 0.303 |  |  |  |  |
|  | Snapchat (b) | 0.002 | 0.002 | *F*(1, 32.11) = 1.03*; t*(32.11) = 1.02 | 0.317 |  |  |  |  |
|  | Instagram (w-lead) | -0.001 | 0.001 | *F*(1, 201.12) = 0.75*; t*(201.12) = -0.87 | 0.388 |  |  |  |  |
|  | Tik Tok (w-lead) | -0.004 | 0.002 | *F*(1, 208.95) = 4.29*; t*(208.95) = -2.07 | 0.040 |  |  |  |  |
|  | Messenger (w-lead) | 0.000 | 0.002 | *F*(1, 201.45) = 0.00*; t*(201.45) = -0.02 | 0.981 |  |  |  |  |
|  | Snapchat (w-lead) | 0.001 | 0.001 | *F*(1, 200.83) = 0.25*; t*(200.83) = 0.5 | 0.617 |  |  |  |  |
|  | Instagram (w-lag) | 0.003 | 0.001 | *F*(1, 201.17) = 3.89*; t*(201.17) = 1.97 | 0.050 |  |  |  |  |
|  | Tik Tok (w-lag) | -0.001 | 0.002 | *F*(1, 203.71) = 0.1*; t*(203.71) = -0.32 | 0.751 |  |  |  |  |
|  | Messenger (w-lag) | -0.005 | 0.002 | *F*(1, 204.46) = 8.33*; t*(204.46) = -2.89 | 0.004 |  |  |  |  |
|  | Snapchat (w-lag) | -0.001 | 0.001 | *F*(1, 203.06) = 1.32*; t*(203.06) = -1.15 | .0253 |  |  |  |  |
|  | **Recovery** |  |  |  |  |  |  |  |  |
|  | Intercept | 4.642 | .286 | *F*(1, 31.25) = 263.82*; t*(31.25) = 16.24 | <0.001 | 0.411 | 0.117 | 3.499 | <0.001 |
|  | Instagram (b) | 0.006 | 0.003 | *F*(1, 31.2) = 2.99*; t*(31.07) = 1.73 | 0.094 |  |  |  |  |
|  | TikTok (b) | 0.004 | 0.004 | *F*(1, 32.75) = 0.90; *t*(32.75) = 0.95 | 0.350 |  |  |  |  |
|  | Messenger (b) | 0.000 | 0.005 | *F*(1, 31.67) = 0.00*; t*(31.67) = -0.04 | 0.968 |  |  |  |  |
|  | Snapchat (b) | -0.001 | 0.002 | *F*(1, 32.06) = 0.34*; t*(32.06) = -0.58 | 0.565 |  |  |  |  |
|  | Instagram (w-lead) | -0.001 | 0.001 | *F*(1, 202.4) = 0.16*; t*(202.4) = -0.40 | 0.687 |  |  |  |  |
|  | Tik Tok (w-lead) | -0.006 | 0.002 | *F*(1, 209.72) = 10.12*; t*(209.72) = -3.18 | 0.002 |  |  |  |  |
|  | Messenger (w-lead) | 0.000 | 0.002 | *F*(1, 202.39) = 0.08*; t*(202.39) = -0.28 | 0.782 |  |  |  |  |
|  | Snapchat (w-lead) | 0.000 | 0.001 | *F*(1, 201.82) = 0.03*; t*(201.82) = -0.17 | 0.862 |  |  |  |  |
|  | Instagram (w-lag) | 0.001 | 0.001 | *F*(1, 202.29) = 0.59*; t*(202.29) = 0.77 | 0.444 |  |  |  |  |
|  | Tik Tok (w-lag) | -0.001 | 0.002 | *F*(1, 205.14) = 0.46*; t*(205.14) = -0.68 | 0.500 |  |  |  |  |
|  | Messenger (w-lag) | 0.001 | 0.002 | *F*(1, 205.48) = 0.24*; t*(205.48) = 0.49 | 0.628 |  |  |  |  |
|  | Snapchat (w-lag) | 0.000 | 0.001 | *F*(1, 204.19) = 0.00*; t*(204.19) = -0.02 | 0.987 |  |  |  |  |
|  | **Stress** |  |  |  |  |  |  |  |  |
|  | Intercept | 3.231 | .326 | *F*(1, 31.29) = 98.42*; t*(31.29) = 9.92 | <0.001 | 0.527 | 0.152 | 3.455 | <0.001 |
|  | Instagram (b) | -0.005 | 0.004 | *F*(1, 30.97) = 1.45*; t*(30.97) = -1.20 | 0.238 |  |  |  |  |
|  | TikTok (b) | -0.001 | 0.005 | *F*(1, 32.59) = 0.02*; t*(32.59) = -0.15 | 0.882 |  |  |  |  |
|  | Messenger (b) | 0.002 | 0.006 | *F*(1, 32.15) = 0.14*; t*(32.15) = 0.37 | 0.711 |  |  |  |  |
|  | Snapchat (b) | -0.001 | 0.003 | *F*(1, 32.08) = 0.04*; t*(32.08) = -0.21 | 0.836 |  |  |  |  |
|  | Instagram (w-lead) | 0.002 | 0.002 | *F*(1, 205.4) = 1.61*; t*(205.4) = 1.27 | 0.206 |  |  |  |  |
|  | Tik Tok (w-lead) | 0.005 | 0.002 | *F*(1, 213.42) = 5.66*; t*(213.42) = 2.38 | 0.018 |  |  |  |  |
|  | Messenger (w-lead) | -0.003 | 0.002 | *F*(1, 207.18) = 1.97*; t*(207.18) = -1.41 | 0.162 |  |  |  |  |
|  | Snapchat (w-lead) | 0.000 | 0.001 | *F*(1, 204.88) = 0.00*; t*(204.88) = -0.05 | 0.959 |  |  |  |  |
|  | Instagram (w-lag) | -0.001 | 0.002 | *F*(1, 205.3) = 0.70*; t*(205.3) = -0.83 | 0.405 |  |  |  |  |
|  | Tik Tok (w-lag) | 0.004 | 0.002 | *F*(1, 208.15) = 2.60*; t*(208.15) = 1.61 | 0.108 |  |  |  |  |
|  | Messenger (w-lag) | 0.002 | 0.002 | *F*(1, 209.39) = 0.59*; t*(209.39) = 0.77 | 0.444 |  |  |  |  |
|  | Snapchat (w-lag) | 0.000 | 0.001 | *F*(1, 207.4) = 0.02*; t*(207.4) = 0.14 | 0.890 |  |  |  |  |

*Notes: * B = Unstandardized beta coefficient. Beta = Standardized beta coefficient. Estimate = Variance Estimate. (b) = between subjects. (w) = within subjects.*

**Supplementary Table S(2)**

*Parameters of the mixed model analyses for energetic arousal, calmness, and valence.*

|  | **Outcome** | **Fixed B** | ***SE*** | ***F* (df)*; t* (df)** | ***p*** | **Random Estimate** | ***SD*** | **Wald-*Z*** | ***p*** |
| --- | --- | --- | --- | --- | --- | --- | --- | --- | --- |
|  | **Energetic Arrousal** |  |  |  |  |  |  |  |  |
|  | Intercept | 7.880 | 0.677 | *F*(1, 28.5) = 135.39*; t*(28.5) = 11.636 | <0.001 | 1.825 | 0.688 | 2.653 | 0.008 |
|  | Instagram (b) | 0.012 | 0.008 | *F*(1, 28.04) = 2.22*; t*(28.04) = 1.49 | 0.147 |  |  |  |  |
|  | TikTok (b) | 0.005 | 0.010 | *F*(1, 31.87) = 0.25*; t*(31.87) = 0.50 | 0.620 |  |  |  |  |
|  | Messenger (b) | -0.007 | 0.012 | *F*(1, 30.35) = 0.35*; t*(30.35) = -0.59 | 0.558 |  |  |  |  |
|  | Snapchat (b) | -0.008 | 0.006 | *F*(1, 31.5) = 2.25*; t*(31.5) = -1.5 | 0.144 |  |  |  |  |
|  | Instagram (w-lead) | -0.001 | 0.005 | *F*(1, 205.58) = 0.06*; t*(205.58) = -0.25 | 0.804 |  |  |  |  |
|  | Tik Tok (w-lead) | -0.006 | 0.007 | *F*(1, 219.63) = 0.69*; t*(219.63) = -0.83 | 0.408 |  |  |  |  |
|  | Messenger (w-lead) | 0.001 | 0.006 | *F*(1, 206.17) = 0.00*; t*(206.17) = 0.09 | 0.925 |  |  |  |  |
|  | Snapchat (w-lead) | -0.003 | 0.004 | *F*(1, 204.31) = 0.66*; t*(204.31) = -0.81 | 0.419 |  |  |  |  |
|  | Instagram (w-lag) | 0.007 | 0.005 | *F*(1, 205.35) = 2.12*; t*(205.35) = 1.46 | 0.146 |  |  |  |  |
|  | Tik Tok (w-lag) | -0.002 | 0.008 | *F*(1, 210.61) = 0.09*; t*(210.61) = -0.3 | 0.768 |  |  |  |  |
|  | Messenger (w-lag) | 0.002 | 0.007 | *F*(1, 214.38) = 0.06*; t*(214.38) = 0.24 | 0.813 |  |  |  |  |
|  | Snapchat (w-lag) | -0.001 | 0.004 | *F*(1, 208.90) = 0.03*; t*(208.90) = -0.16 | 0.870 |  |  |  |  |
|  | **Calmness** |  |  |  |  |  |  |  |  |
|  | Intercept | 8.200 | 0.663 | *F*(1, 31.33) = 152.96*; t*(31.33) = 12.37 | <0.001 | 2.153 | 0.632 | 3.406 | <0.001 |
|  | Instagram (b) | 0.018 | 0.008 | *F*(1, 31.01) = 5.27*; t*(31.01) = 2.3 | 0.029 |  |  |  |  |
|  | TikTok (b) | 0.004 | 0.010 | *F*(1, 32.78) = 0.14*; t*(32.78) = 0.38 | 0.710 |  |  |  |  |
|  | Messenger (b) | 0.012 | 0.011 | *F*(1, 31.87) = 1.04*; t*(31.87) = 1.02 | 0.316 |  |  |  |  |
|  | Snapchat (b) | -0.007 | 0.005 | *F*(1, 32.25) = 1.64*; t*(32.25) = -1.28 | 0.209 |  |  |  |  |
|  | Instagram (w-lead) | 0.001 | 0.003 | *F*(1, 205.46) = 0.06*; t*(205.46) = 0.25 | 0.800 |  |  |  |  |
|  | Tik Tok (w-lead) | -0.001 | 0.005 | *F*(1, 214.13) = 0.06*; t*(214.13) = -0.24 | 0.813 |  |  |  |  |
|  | Messenger (w-lead) | -0.004 | 0.004 | *F*(1, 205.71) = 1.05*; t*(205.71) = -1.02 | 0.307 |  |  |  |  |
|  | Snapchat (w-lead) | -0.003 | 0.003 | *F*(1, 204.97) = 1.39*; t*(204.97) = -1.18 | 0.240 |  |  |  |  |
|  | Instagram (w-lag) | -0.001 | 0.003 | *F*(1, 205.34) = 0.19*; t*(205.34) = -0.43 | 0.666 |  |  |  |  |
|  | Tik Tok (w-lag) | -0.005 | 0.005 | *F*(1, 208.72) = 0.79*; t*(208.72) = -0.89 | 0.375 |  |  |  |  |
|  | Messenger (w-lag) | -0.003 | 0.005 | *F*(1, 209.49) = 0.39*; t*(209.49) = -0.63 | 0.532 |  |  |  |  |
|  | Snapchat (w-lag) | 0.001 | 0.003 | *F*(1, 207.72) = 0.10*; t*(207.72) = 0.32 | 0.750 |  |  |  |  |
|  | **Valence** |  |  |  |  |  |  |  |  |
|  | Intercept (b) | 8.615 | 0.542 | *F*(1, 31.16) = 252.31*; t*(31.16) = 15.88 | <0.001 | 1.356 | 0.423 | 3.206 | 0.001 |
|  | Instagram (b) | 0.013 | 0.006 | *F*(1, 30.97) = 4.45*; t*(30.97) = 2.11 | 0.043 |  |  |  |  |
|  | TikTok (b) | -0.003 | 0.008 | *F*(1, 33.26) = 0.13*; t*(33.26) = -0.36 | 0.718 |  |  |  |  |
|  | Messenger (b) | 0.014 | 0.009 | *F*(1, 32.1) = 2.39*; t*(32.1) = 1.55 | 0.132 |  |  |  |  |
|  | Snapchat (b) | -0.004 | 0.004 | *F*(1, 32.64) = 0.93*; t*(32.64) = -0.97 | 0.341 |  |  |  |  |
|  | Instagram (w-lead) | -0.003 | 0.003 | *F*(1, 202.83) = 1.02*; t*(202.83) = -1.01 | 0.313 |  |  |  |  |
|  | Tik Tok (w-lead) | -0.005 | 0.005 | *F*(1, 212.12) = 1.28*; t*(212.12) = -1.13 | 0.259 |  |  |  |  |
|  | Messenger (w-lead) | 0.001 | 0.004 | *F*(1, 202.35) = 0.15*; t*(202.35) = 0.39 | 0.700 |  |  |  |  |
|  | Snapchat (w-lead) | -0.002 | 0.003 | *F*(1, 201.27) = 0.75*; t*(201.27) = -0.87 | 0.387 |  |  |  |  |
|  | Instagram (w-lag) | 0.005 | 0.003 | *F*(1, 201.77) = 2.03*; t*(201.77) = 1.42 | 0.156 |  |  |  |  |
|  | Tik Tok (w-lag) | -0.005 | 0.005 | *F*(1, 205.75) = 1.00*; t*(205.75) = -1.00 | 0.318 |  |  |  |  |
|  | Messenger (w-lag) | -0.001 | 0.005 | *F*(1, 207.34) = 0.03*; t*(207.34) = -0.17 | 0.869 |  |  |  |  |
|  | Snapchat (w-lag) | 0.001 | 0.003 | *F*(1, 204.57) = 0.05*; t*(204.57) = 0.21 | 0.832 |  |  |  |  |

*Notes: * B = Unstandardized beta coefficient. Beta = Standardized beta coefficient. Estimate = Variance Estimate. (b) = between subjects. (w) = within subjects*

**Supplementary Table S(1)**

*Parameters of the mixed model analyses for sleep, recovery, and stress.*

*Notes: * B = Unstandardized beta coefficient. Beta = Standardized beta coefficient. Estimate = Variance Estimate. (b) = between subjects. (w) = within subjects.*

**Supplementary Table S(2)**

*Parameters of the mixed model analyses for energetic arousal, calmness, and valence.*

*Notes: * B = Unstandardized beta coefficient. Beta = Standardized beta coefficient. Estimate = Variance Estimate. (b) = between subjects. (w) = within subjects.*

**Supplementary Information S(3)**

**Results of the robustness analyses**

**Gender**

For lead model parameters, significant results in the were found for TikTok and recovery (*F*(1, 233.60)=13.26, *p* <0.001), stress (*F*(1,236.96) = 8.38, *p* = 0.004), and sleep (*F*(1, 234.20) = 8.32, *p* = 0.004) on an intraindividual level. For lag model parameters, significant results in the were found for TikTok and recovery stress (*F*(1, 227.51) = 7.57, *p* = 0.006) on an intraindividual level. Further, there was a significant positive relationship between sleep and Instagram (*F*(1, 220.16) = .4.06, *p* = 0.045) and a negative relationship between messengers and sleep (*F*(1, 220.95) = 8.14, *p* = 0.028)

Interindividual effects were found for Instagram and calmness (*F*(1, 30.40) = 6.20, *p* = 0.019) and valence (*F*(1, 30.16) = 4.61, *p* = 0.040). No other significant effects were found.

**Team vs individual sports**

For lead model parameters, significant results in the were found for TikTok and recovery (*F*(1, 233.67)=13.54, *p* < 0.001), stress (*F*(1, 237.09) = 8.51, *p* = 0.004), and sleep (*F*(1, 234.83) = 8.86, *p* = 0.003) on an intraindividual level. For lag model parameters, significant effects were found for stress and Tiktok (*F*(1, 227.62) = 7.38, *p* = 0.007). Additionally, sleep was related positively to intraindividual usage of Instagram (*F*(1, 219.89) = 4.06, *p* = 0.045) and negatively to intraindividual usage of messengers (*F*(1, 220.80) = 8.11, *p* < 0.001). Interindividual effects were found for Instagram and calmness (*F*(1, 29.89) = 4.63, *p* = 0.040) and valence (*F*(1, 29.75) = 4.38, *p* = 0.045). No other significant effects were found.
